# Chemical Variation in a Dominant Tree Species: Population Divergence, Selection and Genetic Stability across Environments

**DOI:** 10.1371/journal.pone.0058416

**Published:** 2013-03-20

**Authors:** Julianne M. O’Reilly-Wapstra, Alison M. Miller, Matthew G. Hamilton, Dean Williams, Naomi Glancy-Dean, Brad M. Potts

**Affiliations:** 1 School of Plant Science, University of Tasmania, Hobart, Tasmania, Australia; 2 Forestry Tasmania, Hobart, Tasmania, Australia; 3 National Centre for Future Forest Industries, University of Tasmania, Hobart, Tasmania, Australia; Centro de Investigación y de Estudios Avanzados, Mexico

## Abstract

Understanding among and within population genetic variation of ecologically important plant traits provides insight into the potential evolutionary processes affecting those traits. The strength and consistency of selection driving variability in traits would be affected by plasticity in differences among genotypes across environments (G×E). We investigated population divergence, selection and environmental plasticity of foliar plant secondary metabolites (PSMs) in a dominant tree species, *Eucalyptus globulus*. Using two common garden trials we examined variation in PSMs at multiple genetic scales; among 12 populations covering the full geographic range of the species and among up to 60 families within populations. Significant genetic variation in the expression of many PSMs resides both among and within populations of *E. globulus* with moderate (e.g., sideroxylonal A h^2^op = 0.24) to high (e.g., macrocarpal G h^2^op = 0.48) narrow sense heritabilities and high coefficients of additive genetic variation estimated for some compounds. A comparison of Qst and Fst estimates suggest that variability in some of these traits may be due to selection. Importantly, there was no genetic by environment interaction in the expression of any of the quantitative chemical traits despite often significant site effects. These results provide evidence that natural selection has contributed to population divergence in PSMs in *E. globulus*, and identifies the formylated phloroglucinol compounds (particularly sideroxylonal) and a dominant oil, 1,8-cineole, as candidates for traits whose genetic architecture has been shaped by divergent selection. Additionally, as the genetic differences in these PSMs that influence community phenotypes is stable across environments, the role of plant genotype in structuring communities is strengthened and these genotypic differences may be relatively stable under global environmental changes.

## Introduction

Phenotypic variation in traits across the geographic range of a species can be the result of a variety of environmental effects and/or genetic processes such as local adaptation and drift [1], [2]. A key interest for many ecologists and evolutionary biologists is to unravel these genetic processes to better understand evolutionary forces affecting ecologically relevant traits [3]. A useful approach to achieve this is to investigate how phenotypic variation is partitioned at different genetic levels, such as among and within populations [4], [5], [6]. Among population variation in traits may indicate population divergence in trait evolution while estimation of within population genetic parameters can be used to understand how these traits may respond to selection**.** For example, narrow sense heritability is a useful within population genetic parameter to estimate the proportion of phenotypic variance that is due to additive effects (and thus is the variance selection can act upon). This matched with the coefficient of additive genetic variance (the additive genetic variance standardised by the trait mean) to assess the ‘evolvability’ of a trait, provides a clear methodology for understanding the evolutionary potential of traits [7], [8].

Plant/herbivore systems represent a model approach to investigate patterns of evolutionary population divergence across the geographic range of a species and the evolutionary processes affecting relevant traits within populations. Major drivers of plant/herbivore interactions are plant secondary metabolites (PSMs; compounds produced as a result of plant secondary metabolism) [9], [10], [11], [12]. Many studies have investigated the evolutionary processes affecting the expression of PSMs in plants [13], [14], [15], however, relatively few have taken a hierarchical genetic approach to better understand the genetic basis of natural variation in these compounds [5]. In addition, the strength and consistency of evolutionary pressures in driving variability in PSMs would be affected by variation in the plasticity of genotypes in the way they respond to environmental variation (G×E) [16], and these traits may also be affected by correlated responses to selection [17]. Consequently, studies that take a hierarchical genetic approach, combined with genotype by environment manipulations and an understanding of how multiple PSM traits are genetically correlated, are beneficial to understand the multiple factors that lead to variation in the evolution of PSMs in plant/herbivore systems.

*Eucalyptus globulus* is a dominant tree species of ecological significance in south-eastern Australia, and is rich in foliar PSMs including terpenes and phenolic based compounds such as formylated phloroglucinol compounds (FPCs). These compounds affect rates of herbivory by many marsupial herbivores and structure foliar arthropod communities [18], [19], [20], [21]. The quantitative genetic expression of some of these traits in *E. globulus* is well understood, however, previous work has focused on among population genetic variation [18], [19], [22]. Little is known about the levels of within population level genetic variation (and hence ‘evolvability’) of these key plant traits. Even less is known about how variable genotypes are in the expression of PSMs at both the within and among population levels across different environments [23] and how these chemical traits relate to growth or fitness traits in the species [24].

We advance previous research on broad-scale geographic genetic variation in PSMs in *E. globulus* [22], [25] by taking a within population, family (sibling) level approach to better understand genetic variation in key PSMs across multiple genetic scales (by comparing among and within population variation) and to better understand variation in these traits at the scale at which natural selection operates (within populations). Due to our addition of family pedigrees we can estimate quantitative genetic parameters including narrow sense heritability, coefficient of additive genetic variation and compare estimates of quantitative (Qst) versus neutral (Fst) genetic variation. All of these estimates provide important information on a traits ability to respond to selection [1], [7], [26]. We also examine the inter-trait correlations between these compounds across the different levels of the genetic hierarchy (population and family) to investigate if within population genetic correlations relate to among population patterns of divergence in these traits. We examine how these traits relate to plant growth and browsing damage in the field and importantly, we examine the genetic-based plasticity of PSMs at these different genetic scales, across different environments.

## Materials and Methods

### Ethics statement

No permits or approvals were required for this field study as only small samples of an abundant tree species were taken. Effects to individual and ecosystem health were negligible. The land accessed is not protected. No protected species were sampled.

### Genetic pedigree of *E. globulus*

*Eucalyptus globulus* has been classified into a genetic hierarchy of 13 geographically and genetically distinct races and 20 sub-races across its full geographic range, based on differences in 35 different morphological traits assessed in a range of common environment trials [27]. Families within sub-races are offspring from a single mother tree [27], [28].

### Trial design, sampling and browse assessment

In this study we utilised two common environment field trials established with progeny of known family pedigree and representative of a large proportion of *E. globulus* sub-races. Foliage was collected from these progeny trials located at Togari (40° 57′ S, 144° 55′ E, 90 m asl) and Salmon River (41° 02′ S, 144° 49′ E, 100 m asl) in north-west Tasmania. Both field trials were established in August 2005 by Forestry Tasmania. Sites differed markedly in their soil profiles. Salmon River was yellow brown mottled clay on Precambarian mudstone whereas Togari was red brown clay on Cambrian inter-layered mudstone, siltstone and sandstone. Each trial comprised plants grown from open-pollinated seed collected from 310 (Salmon River) and 244 (Togari) families collected from throughout the native, natural distribution of *E. globulus* (see Dutkowski & Potts [27] for details). Furthermore, seedlots from breeding programmes and commercial seed-orchards were included in the trials. Each trial was a randomised incomplete block design with each family represented as a single-tree plot. Salmon River (SR) comprised 20 replicates of 15 incomplete blocks per replicate while Togari (TO) comprised 16 replicates of 12 incomplete blocks.

**Figure 1:**
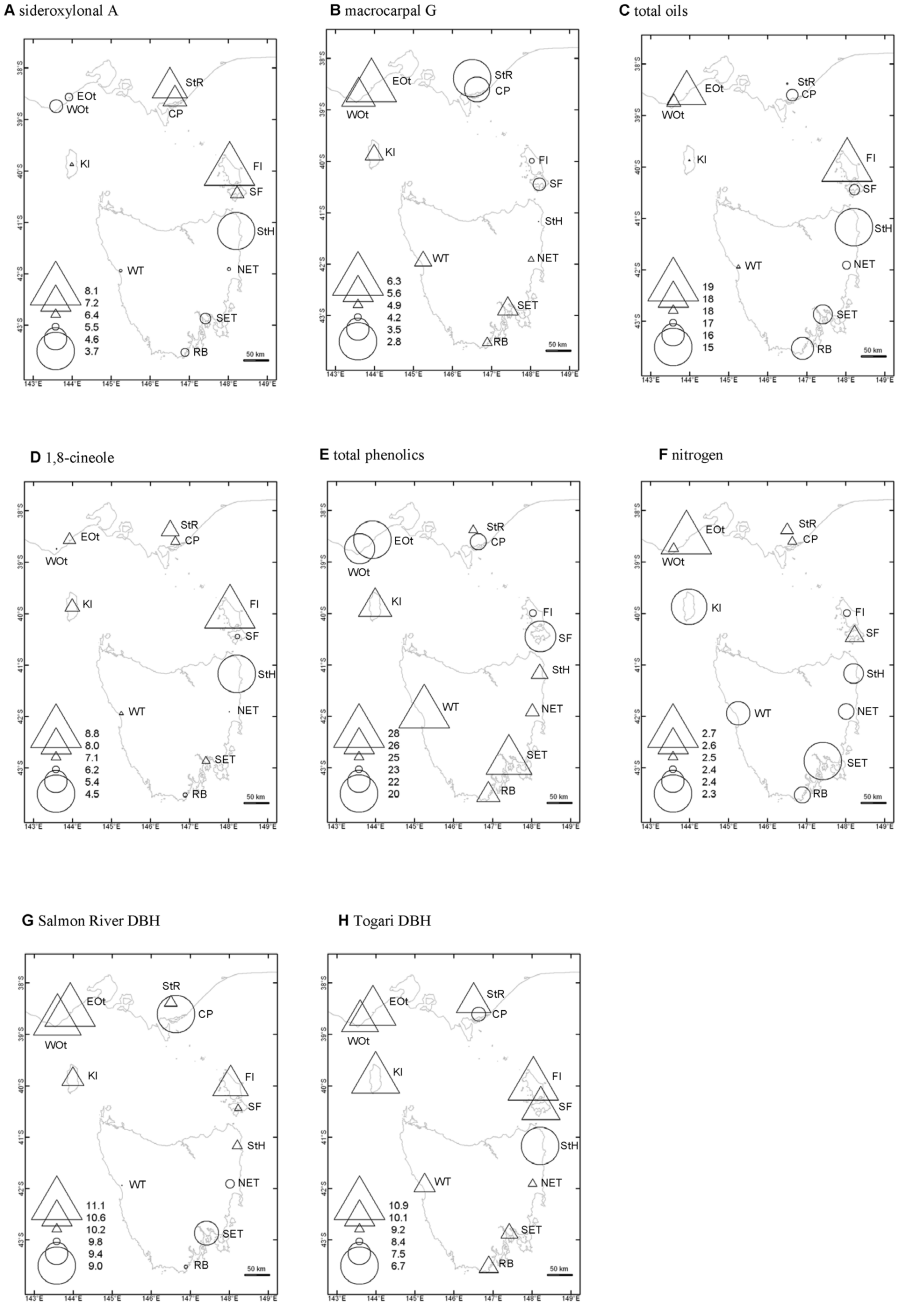
Genetic variation in seven traits amongst open-pollinated families of *Eucalyptus globulus* grown in two Tasmanian field trials. The geographical source of each locality in Victoria, Tasmania and the Bass Strait islands is shown. Symbols represent the genetic-based variation in foliar concentration of six chemical constituents (A – F) and DBH (G, H) as shown in the left of the figures. *Triangles* indicate relatively high values, with larger triangles the highest values. *Circles* indicate relatively low values, with larger circles the lowest values. Results averaged across two common environment field trials for the chemicals; separate figures for each site for DBH due to a significant site*sub-race interaction (Table 2). Sub-race codes as in Table 1.

Juvenile foliage was harvested from a subset of trees at each site in March 2006. Two healthy leaves were taken from each individual tree. Foliage was selected from the northeast side, half way up the height of the seedlings (seedlings were approximately one metre tall), three leaf pairs back from branch tips. This ensured that fully expanded juvenile leaves (but not yet entering senescence) were sampled and that leaves sampled were the same age across all plants. Trees harvested from Salmon River represented 60 families from 12 sub-races while samples were taken from trees representing 53 families from the same 12 sub-races at Togari. These were collected across 20 and 15 replicates from Salmon River and Togari, respectively. In total 749 samples were collected from the two sites (Table 1). Sub-race locations are shown in Figure 1. Leaves were placed in cool storage and frozen on return to the laboratory. Leaves were later freeze-dried in preparation for predictive chemical analysis using near infrared reflectance spectroscopy, after which time they were ground in a cyclone mill for wet chemical analysis.

**Table 1:** Number of families and individuals sampled at each hierarchical genetic scale.

		Families		Individuals	
Sub-race	Sub-race code	SR	TO	SR	TO
Eastern Otways	EOt	6	5	41	31
Flinders Island	FI	3	3	19	25
Southern Furneaux	SF	6	4	31	31
King Island	KI	5	5	42	28
St Helens	StH	8	5	43	34
North-eastern Tasmania	NET	4	5	30	33
Recherche Bay	RB	3	3	20	27
South-eastern Tasmania	SET	4	4	16	24
Coastal Plain	CP	6	6	37	41
Strzelecki Ranges	StR	5	5	49	37
Western Otways	WOt	6	5	44	34
Western Tasmania	WT	4	3	19	13

Sub-race codes are used in Figure 1.

*SR* Salmon River common garden site, *TO* Togari common garden site.

Mammal browsing damage was scored at Togari in March 2006. Trees at Salmon River were not browsed. Browsing damage was scored as percentage of total foliage removed from the whole plant on a scale from 0 to 6, where 0 = 0%, 1 = 1−5%, 2 = 6−25%, 3 = 26−50%, 4 = 51−75%, 5 = 76−95%, and 6 = 96−100%. Due to generally low browsing (∼27% of trees received damage), scores were converted to a binary presence/absence for analysis. Diameter at breast height (DBH; 1.3m) was assessed in February 2010 after 5 years growth to relate browsing and foliar chemical variation with a surrogate measure of tree fitness.

### Chemical analysis

#### Near infrared reflectance spectroscopy

We predicted the concentration of six different chemical constituents in the leaves using near infrared reflectance spectroscopy (NIRS). These constituents were nitrogen, two formylated phloroglucinol compounds (sideroxylonal A and macrocarpal G), total oils, 1,8-cineole (the dominant oil in *E. globulus*) and total phenolics. Near infrared reflectance spectroscopy has been used extensively in ecology and other fields to enable rapid analysis of samples on a scale that would be impractical using conventional laboratory techniques (see Foley *et al.* [29] for a review). For each sample (n =  749) two freeze-dried leaves were scanned with near-infrared light using a Bruker MPA FT-NIR spectrometer coupled to a fibre-optic probe. The tip and the basal region of the adaxial surface of each leaf were scanned, avoiding the midrib and damaged areas. Spectra (expressed as log (1/R)) were collected from 7690–4350 cm^−1^ (1300–2300nm) at a resolution of 4 cm^−1^. The resulting four spectra taken from the two leaves were averaged.

#### Near infrared reflectance spectroscopy model development and predictions

Near infrared reflectance spectroscopy models were developed to predict the concentration of all chemical constituents. Prior to model development, principal component analysis (PCA) was carried out on the spectra of all samples, to enable selection of a subset (n = 100; 50 samples per site) of samples for wet chemical analysis (see below) for model validation purposes. These selected samples were representative of the spectral variation present in the entire sample set. Half of these samples (n = 50) were added to existing NIRS models [30] and these models were re-developed to predict the concentration of the samples used in this study. The other half of the samples (n = 50) were used as an independent validation set of the predictive models. The oil models were developed using only the samples used for wet chemical analysis in this current study (that is, not built from existing models). All models were developed using internal cross validation to avoid overfitting. Models showed a close relationship between wet chemistry and predicted values, both within the calibration set and upon independent validation. Internal cross validation results for the NIRS models ranged from r^2^ 0.66–0.90 (SEP: 0.22–5.21) for the six chemical traits; the external validation r^2^ ranged from 0.69–0.86 (SEP: 1.29–5.05).

#### Wet chemistry

Wet chemical analysis was carried out on 50 samples per site. Total oils and 1,8-cineole were assayed by gas chromatography-mass spectrometry (GCMS). The method was modified from that reported in O'Reilly-Wapstra et al. [19] whereby freeze-dried ground samples were used for extraction and results were expressed as mg g DM^−1^ equivalents of cineole using a 1,8-cineole standard. Total phenolics were assayed with a modified Prussian blue assay for total phenolics using gallic acid standards [31] after extraction following the method outlined in Hagerman [32]. Using a gallic acid standard, total phenolics concentration was expressed as mg g DM^−1^ equivalents of gallic acid. Sideroxylonal A and macrocarpal G were assayed by high performance liquid chromatography (HPLC) following Wallis & Foley [33]. By using a pure standard of sideroxylonal A, results were expressed as mg g DM^−1^. Results for macrocarpal G concentration were expressed as mg g DM^−1^ equivalents of macrocarpal A (using a macrocarpal A standard). Freeze dried ground leaf samples were re-ground to a fine powder using a ball grinder (Retsch MM200) and analysed for nitrogen using an elemental analyser (Thermo Finnigan EA 1112 Series).

### Statistical analysis

Statistical analyses were conducted using ASReml™ [34] and SAS 9.2 [35]. For all statistical tests, residuals were checked for homoscedasticity and normality, and transformations were performed where required [36]. Total phenolics data were log transformed. The browsing score was treated as a binary trait and analysed using a probit link function.

### Genetic, environment and G×E effects on traits

To examine the variation in foliage chemistry across the two common environment trials, among sub-races and families within sub-races, as well as genetic-by-environment interactions, using only families from the native range (that is, not including the breeding programme seedlots) we fitted the following model with the PROC MIXED procedure of SAS:

Y = μ + SITE + SUBRACE + SITE*SUBRACE + REP + FAM (SUBRACE) + SITE*FAM (SUBRACE) + RESIDUAL

whereby, Y is the phenotypic observation, μ is the mean, SITE is the fixed site effect, SUBRACE is the fixed sub-race effect, SITE*SUBRACE is the fixed site by sub-race interaction effect, *REP* is the random replicate within site effect, *FAM (SUBRACE)* is the random family within sub-race effect, SITE*FAM (SUBRACE) is the random site by family within sub-race interaction effect, *RESIDUAL* is the residual. Fixed effects of SITE and SUBRACE, including the interaction term, were effectively our ‘treatment’ effects.

Discriminant analysis separating sub-races was conducted on a data set comprising the native-forest family means (calculated across sites) for the six chemical components using the PROC DISCRIM procedure of SAS. Mahalanobis distances among sub-races and their significance levels were calculated and summarised by UPGMA clustering undertaken with PROC CLUSTER.

Genetic parameters for each chemical constituent and DBH were estimated for each trial using the following univariate linear model:

Y = μ + GROUP + SUBRACE + REP + FAM (SUBRACE)_native_ + FAM_other_ + RESIDUAL_native_ + RESIDUAL_other_

where Y is the phenotypic observation, μ is the mean, GROUP is the fixed population (either native forest progeny or other progeny from breeding programmes and commercial seed-orchards) effect, SUBRACE is the fixed native-forest sub-race effect, *REP* is the random replicate effect, *FAM (SUBRACE)_native_* is the random family effect (nested within sub-race) for the native forest progeny, *FAM_other_* is the family effect for the non-native progeny, that is, seedlots from breeding programmes and commercial seed-orchards, *RESIDUAL_native,_* is the residuals for the native progeny, and *RESIDUAL_other_* is the residuals for the non-native progeny. The utilisation of all available data (native forest progeny and other progeny from breeding programmes and commercial seed-orchards) in the analysis of individual sites allowed the effect of *REP* to be more precisely estimated, but only the results from native forest progeny are presented.

### Additive genetic variance and heritability estimates

Due to mixed mating systems in eucalypts, additive variance (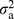), was estimated from open pollinated family variances. The use of open pollinated seed to estimate additive variances is common in tree breeding programmes [26] where assumptions are made that there are no non-additive effects (maternal, dominance or epistasis) and that there is homogeneous outcrossing rates [37], [38]. In *E. globulus*, maternal effects are minimal [39] and studies comparing genetic correlations between open pollinated and control pollinated estimates of additive genetic effects derived from a factorial crossing scheme for frost resistance [40], fungal pathogen damage [41] and other traits summarised in [42], show strong correlations between the open pollinated and controlled crosses. Consequently, we make the assumption of no non-additive effects for our analyses. For the native-forest families, the mean, additive variance (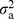), phenotypic variance (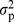), open-pollinated narrow-sense heritability (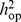) and coefficient of additive genetic variance (*CV*_a_) [7] for each trait were estimated from univariate analysis as follows:
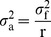


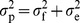


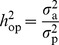


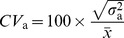



where 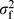 is the family within sub-race variance [from *FAM (SUBRACE)_native_*]; 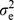 is the residual variance (from *RESIDUAL_native_*); r is the coefficient of relationship, fixed to equal 0.4 to account for an assumed selfing rate of 30% [43]; and 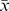 is the trial mean. Standard errors of parameters were estimated from the average information matrix, using a standard truncated Taylor series approximation [34]. A ‘one-tailed’ likelihood ratio test was used to test whether the replicate and family within sub-race variance for each trait and site was significantly greater than zero [34]. The replicate term was dropped from the linear model for traits/sites for which the replicate variance was not significantly different from zero.

### Phenotypic and genetic trait correlations

Bivariate analyses were undertaken for each trait to estimate pair-wise inter-site within sub-race family correlations (which were assumed to be equivalent to additive genetic correlations [44]). The model fitted included the same terms as the univariate model and treated each trait at different sites as a different variate with independent POPN, SUBRACE, *REP* and *RESIDUAL* effects. Furthermore, for each pairwise combination of traits, a multiple-site multiple-trait (i.e. four variates: two traits by two sites) analysis was conducted to estimate inter-trait genetic correlations. The multivariate models used for this purpose combined the aforementioned bivariate models and accounted for covariance between the *REP*, *FAM* and *RESIDUAL* effects for traits within sites. Two-tailed likelihood ratio tests (LRTs) were conducted to test if inter-trait genetic correlations were heterogeneous across sites and to test whether the inter-trait and inter-site genetic correlations were significantly different from zero [45]. One-tailed LRTs were conducted to test genetic correlations against one and minus one. Sub-race correlations were estimated by fitting equivalent models with sub-race fitted as a random, rather than a fixed, term. Phenotypic correlations within sites were estimated in the same way as family correlations by removing all trial design (i.e. *REP*) and genetic terms (i.e. POPN, SUB-RACE and *FAM*) from the model.

### Adaptive divergence amongst sub-races

To estimate the quantitative inbreeding coefficient (*Q*_ST_) [46] for native populations, SUBRACE was fitted as random term in univariate analyses and *Q*_ST_ calculated as follows:
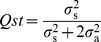



where 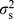 is the sub-race variance and 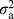 is the additive variance. Following Dutkowski & Potts [47], ‘one-tailed’ likelihood ratio tests [34] were used, to test whether *Q*_ST_ estimates were significantly greater than a published estimate of the mean *F*_ST_ (0.09) which was based on eight microsatellite markers and used a similar suite of *E. globulus* sub-races [48]. More specifically, 10 of the 12 populations used in this current study were used in the *F*st study (where St Helens in this current study is named Humbug Hill in the *F*st study, Flinders Island in the current study is named north and south Flinders island in the *F*st study and finally, Southern Furneaux in the current study is named Cape Barren in the *F*st study). We similarly tested whether the *Q*_ST_ estimates were significantly greater than the maximum of the *F*_ST_ estimate for any microsatellite locus published for this species (0.201; see supplementary material 2 in [49]). *F*_ST_ is an estimate of the population-level inbreeding coefficient for neutral markers, which is assumed to reflect variation brought about by isolation and drift [46], [48]. Assuming comparable mutation rates, significant deviation of *Q*_ST_ from *F*_ST_ is taken as indicative of stabilising (*Q*_ST_<*F*_ST_) or divergent (*Q*_ST_>*F*_ST_) selection having impacted on the genetic architecture of the trait [46], [50]. In our case we were specifically interested in testing for evidence of divergent selection and thus used one-tailed tests.

## Results

Significant differences among sub-races were evident for all chemical constituents (Table 2), with some sub-races containing twice the concentration of the chemical constituents as other sub-races (Figure 1). St Helens had the lowest levels of cineole, total oils and sideroxylonal while at the other extreme, the nearby sub-race, Flinders Island, had the highest level of these constituents (Figure 1). This pattern was reflected in the sub-race positive correlations between cineole and sideroxylonal (Table 3). Several patterns in chemical concentration emerge from Figure 1. Macrocarpal G concentration (Figure 1b) appears highest in the western Victorian sub-races (the Otways), lowest in the eastern Victorian sub-races and intermediate in the Tasmanian populations. Interestingly, patterns for sideroxylonal (Figure 1a) show that the Flinders Island sub-race is similar in concentration to the eastern Victorian sub-races, Southern Furneaux is intermediate and St. Helens on the north-east of Tasmania is lowest, showing a steep cline to north-eastern Tasmania. Additionally, mainland sub-races are lower in concentration of total phenolics compared to Tasmanian sub-races and the opposite pattern is evident for nitrogen. These patterns were reflected in the sub-race inter-trait correlations with a negative correlation between total phenolics and nitrogen (Table 3).

**Table 2:** Results of mixed model analysis of native-forest families examining site, genetic (sub-race and family within sub-race) and interactive effects for each chemical trait and diameter at breast height (DBH).

	sideroxylonal A	macrocarpal G	total oils	cineole	total phenolics	nitrogen	DBH	DBH#
**Fixed**								
Site^1^	38.3***	44.2***	122.1***	53.0***	26.8***	21.0***	1.0	4.8*
Sub-race^2^	11.1***	10.0***	4.3***	8.5***	6.1***	3.5**	4.7***	6.8***
Site*Sub-race^2^	0.9	0.7	0.5	0.7	0.5	0.6	2.1*	7.3***
**Random**								
Rep(Site)	0.8	0.9	2.1*	0.9	1.6	2.0*	0.0	3.6***
Family(Sub-race)	2.4**	3.5***	1.2	2.3*	2.3*	1.5	0.4	5.7***
Site*Family(Sub-race)	0.6	3.0	0.2	3.0	0.4	3.0	0.1	0.6
Residual	17.6***	18.0***	17.6***	18.0***	17.4***	18.1***	17.4***	45.1***

**F (fixed terms) and Z (random terms) values and significance levels are shown.**

1 numerator & denominator DF: 1, 33, except DBH#: 1,34

2 numerator & denominator DF: 11, 37, except DBH#: 12,113

# DBH analysis of the full dataset (not just trees with chemistry data).

* significant at P<0.05; **significant at P<0.01; *** significant at P<0.001.

**Table 3:** Phenotypic (r_p_), additive genetic (r_a_) and sub-race (r_s_) correlations between foliage chemistry, diameter at breast height (*DBH)* and browsing damage.

		sideroxylonal	macrocarpal	total oil	cineole	total phenolics	nitrogen	DBH
macrocarpal	r_p_	-0.23***						
	r_a_	-0.67**						
	r_s_	-0.63						
total oils	r_p_	0.47***	0.45***					
	r_a_	NA	NA					
	r_s_	0.44∧	0.35					
cineole	r_p_	0.66***	0.32***	0.70***				
	r_a_	0.52	0.14	NA				
	r_s_	0.87***	-0.11	0.77***				
total phenolics	r_p_	-0.28***	-0.20***	-0.43***	-0.19***			
	r_a_	-0.40	0.50*	NA	0.27			
	r_s_	-0.04	-0.14	-0.49	-0.51			
nitrogen	r_p_	0.35***	0.04	0.22***	0.07*	-0.48***		
	r_a_	NA	NA	NA	NA	NA		
	r_s_	-0.03	0.04	0.61	0.08	-0.92***		
DBH	TO r_p_	0.11*	0.18***	0.24***	0.19***	-0.33***	0.21***	
	SR r_p_	0.13*	0.20***	0.26***	0.16***	-0.33***	0.14***	
	TO r_a_	0.25	0.23	NA	0.26	-0.31	NA	
	SR r_a_	0.17	0.02	NA	0.48	0.20	NA	
	TOr_s_	0.51	0.22	0.91*	0.77**	-0.33	0.18	
	SR r_s_	0.01	0.57	0.90*	0.16	-0.77*	0.80	
browse	r_p_	-0.07	-0.26***	-0.26***	-0.27***	0.15*	0.14*	-0.30***
	r_a_	NA	NA	NA	NA	NA	NA	NA
	r_s_	-0.42	-0.38	-0.57	-0.73*	-0.43	0.79	0.70*

***To* Togari field site, *SR* Salmon River field site.**

**Correlations for DBH were presented separately for Togari and Salomon River field sites due to genotype by environment interaction at the sub-race level.**

NA = no additive genetic variance

* significant at P<0.05; **significant at P<0.01; *** significant at P<0.001.

∧ =  significance could not be determined using a likelihood ratio test.

Correlations with browse damage are using data from Togari common garden trail only

**Figure 2:**
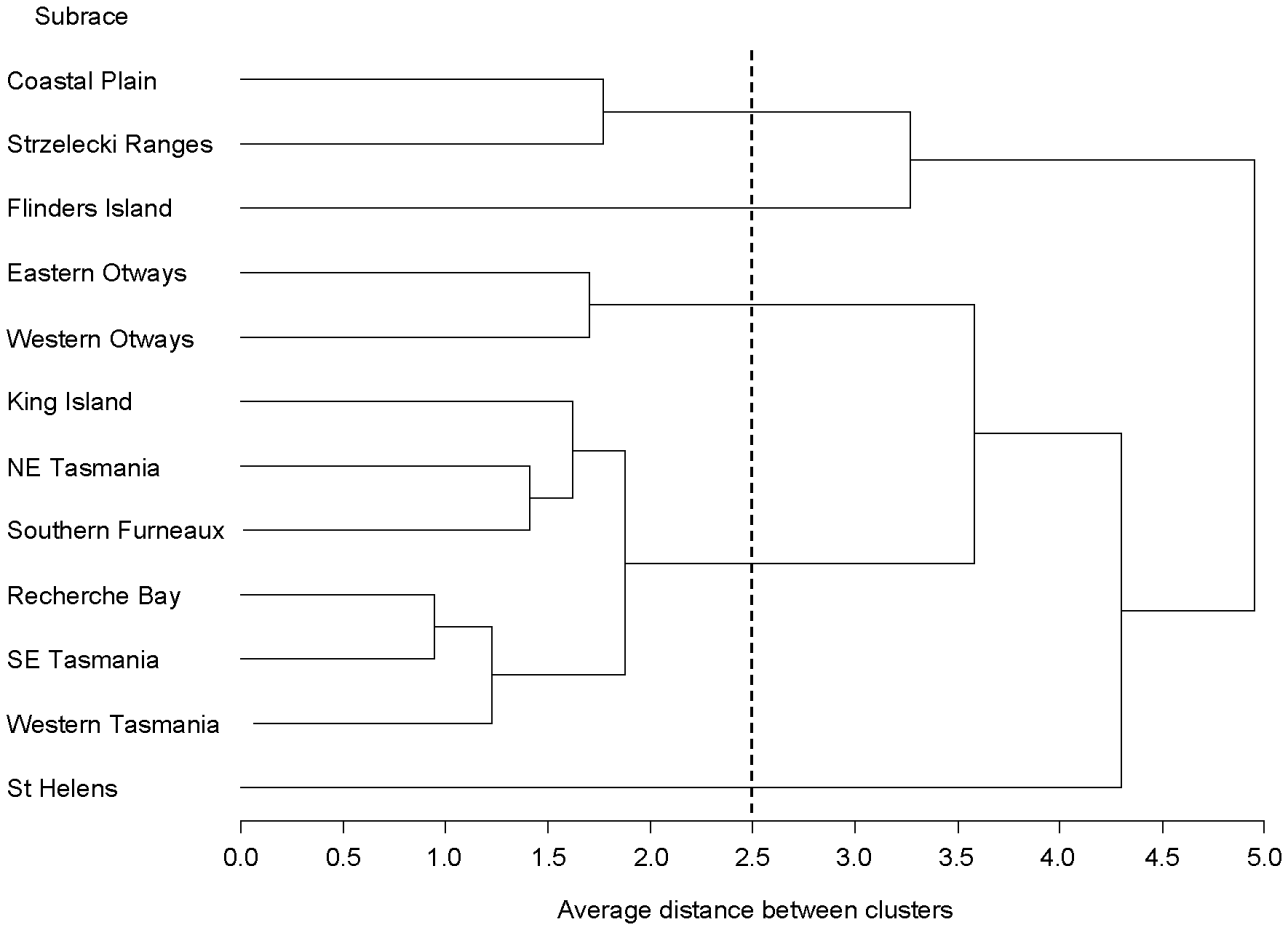
UPGMA clustering of sub-races based on 6 chemical constituents. Dotted line shows where groups are significantly different based on Mahalanobis distances amongst sub-races.

Cluster analysis based on the Mahalanobis distances (Table S1) amongst sub-races resulted in five major sub-race groupings (Figure 2): St Helens and Flinders Island separated independently (although Flinders Island grouped closely to the south-eastern Victoria sub-races), Eastern and Western Otways grouped together, Coastal Plain and Strezleki Ranges grouped together, and the remaining Tasmanian sub-races formed the fifth group. Discriminant analysis identified three significant canonical variates (CVs). CV1 accounted for 51.7% of the variation, CV2 25.8% and CV3 16.4% of the variation, respectively. When CVs were mapped as per Figure 1 (CV maps not shown), CV1 mirrored the distribution of sideroxylonal, CV2 mirrored the distribution of total oils and CV3 mirrored the distribution of nitrogen and total phenolics, indicating that these CVs were strongly influenced by these compounds.

**Figure 3:**
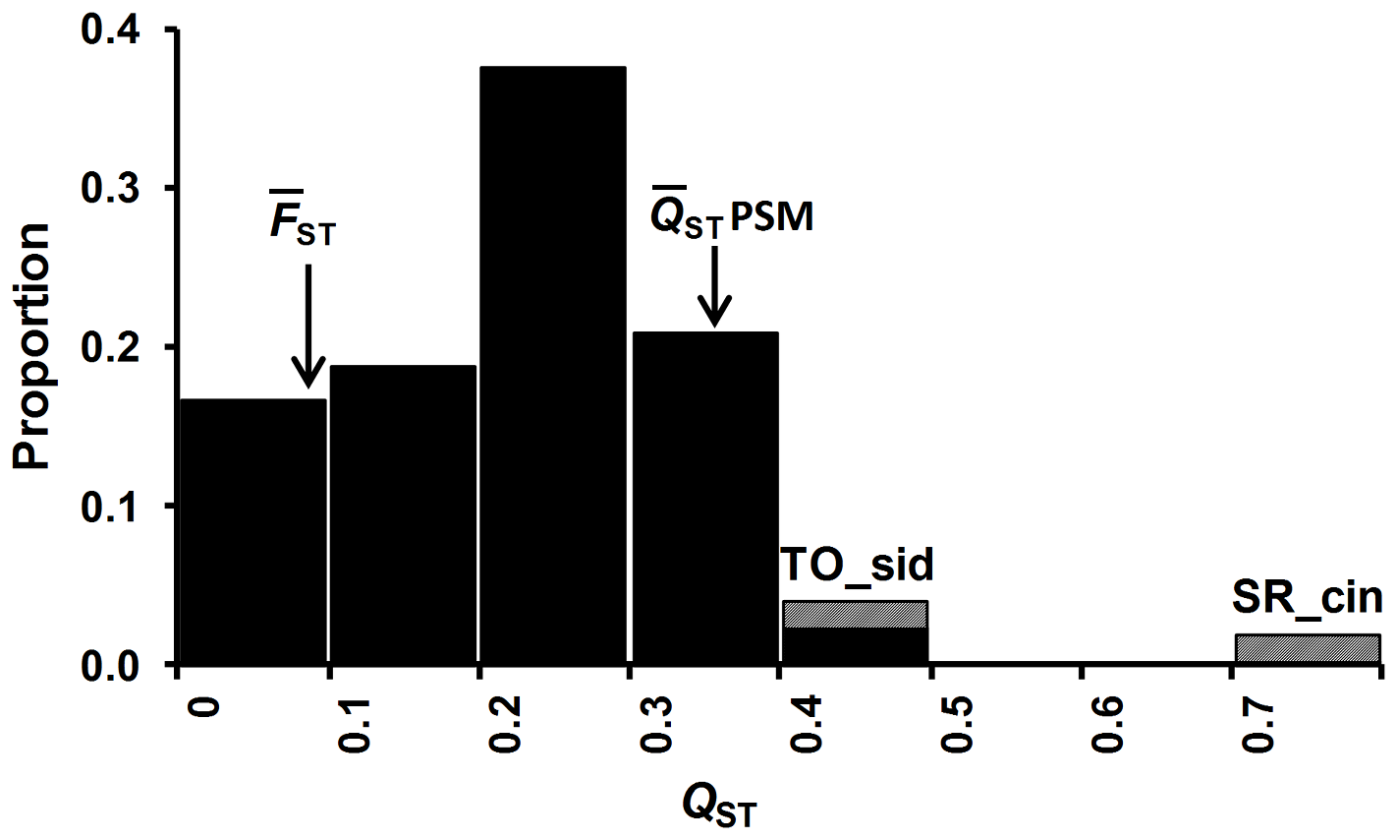
Relative frequency distribution of Qst estimates for *E. globulus*. The Qst data (n = 51) combines published estimates [47] with those from the present study. The position of the outlying values for sideroxylonal (TO_sid) and cineole (SR_cin) are indicated. Arrows show the mean Fst from microsatellite studies (0.09) and the mean Qst for the PSMs from the present study (0.34).

As well as significant among sub-race variation in chemical constituents, there was also significant family within sub-race variation (Table 2) and significant additive genetic variance estimates (Table 4) for the majority of compounds, except for total oils and nitrogen. Open-pollinated narrow sense heritability estimates ranged from 0.07 for nitrogen to 0.48 for macrocarpal. Heritability estimates were not significantly different between sites (p>0.05). The ten Qst estimates for the foliar PSMs ranged from 0.20 to 0.71 and averaged 0.36±0.05. This average was significantly greater than the mean Fst (0.09) for putatively neutral microsatellite loci, (t_9_ =  6.1; 1-tailed P<0.001). Single site Qst estimates for sideroxylonal, macrocarpal, cineole and DBH were also significantly greater than the maximum Fst estimate for individual loci (Table 4). The Qst values for sideroxylonal and cineole were also in the upper tail of the distribution of Qst estimates across diverse traits for *E. globulus* (Figure 3). Several additive genetic correlations between chemical traits were evident (Table 3). A negative additive genetic correlation between sideroxylonal and macrocarpal was significant and there was a significant positive genetic correlation between macrocarpal and total phenolics. Genetic correlations involving oils, nitrogen and browse damage were not examined as there was no family within sub-race variation.

**Table 4:** Means (±SE), quantitative trait differentiation and narrow-sense open-pollinated heritability estimates (h^2^op ) for each trait.

			Between sub-races				Within sub-races				
Trait	Site	Unit	Mean±SE	Qst	SE	P (Qst>Fst)	h^2^op	SE	_CVa_	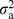	P ( 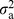 = 0)
sideroxylonal A	SR	mg g DM^−1^	5.45±0.14	0.38	0.18	*	0.24	0.12	16.7	0.830	**
	TO		6.44±0.13	0.43	0.17	**#	0.33	0.14	15.5	1.000	***
macrocarpal G	SR	mg g DM^−1^	4.29±0.10	0.39	0.16	**#	0.35	0.13	19.0	0.663	***
	TO		5.07±0.12	0.30	0.14	*	0.48	0.15	20.4	1.078	***
total oils	SR	mg g DM^−1^	14.34±0.32	0.34	0.29	ns	0.1	0.1	8.6	1.520	ns
	TO		19.43±0.37	0.20	0.22	ns	0.12	0.11	6.8	1.737	ns
cineole	SR	mg g DM^−1^	6.28±0.14	0.71	0.42	** ¥	0.04	0.09	5.9	0.142	ns
	TO		7.45±0.12	0.36	0.18	*	0.25	0.13	11.9	0.798	**
total phenolics	SR	mg g DM^−1^	1.50±0.01	0.27	0.16	ns	0.26	0.12	2.7	0.003	***
	TO		1.46±0.01	0.24	0.14	ns	0.33	0.14	3.4	0.003	***
nitrogen	SR	%	2.34±0.04	0.20	0.26	ns	0.07	0.09	4.6	0.013	ns
	TO		2.55±0.02	0.23	0.25	ns	0.09	0.1	4.9	0.015	ns
DBH	SR	cm	10.17±0.13	0.06	0.04	ns	0.33	0.06	13.8	1.806	***
	TO		9.70±0.22	0.25	0.09	*¥	0.29	0.06	14.6	2.672	***
Browse damage∧	TO	binary	0.22±0.05	0.21	0.15	ns	0.10	0.06	NA	0.108	ns

Differences between sub-races were significant at both sites except for nitrogen at SR (P = 0.055).

h^2^op for each trait was not significantly different between sites (*P* range 0.195–0.893).

All probabilities are based on likelihood ratio tests except the binary browse trait, which was tested using t-tests.

*Qst* quantitative inbreeding coefficient , *SE* standard error, mean *Fst* estimate of neutral marker differentiation, *CV_a_* coefficient of additive genetic variance, 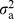 additive genetic variance estimate, *SR* Salmon River *TO* Togari.

∧ Binary (presence/absence) browse damage data were analysed using a probit link function.

* significant at P<0.05; **significant at P<0.01; ***significant at P<0.001

# or ¥ *Qst* is significantly greater than maximum *Fst* estimate published for any microsatellite locus at the P = 0.10 or 0.05 levels, respectively

While there was significant site variation in concentration of all chemical constituents, there were no genotype by environment interactions, i.e. no SITE*SUBRACE, or SITE*FAM effects, in foliage chemistry (Table 2). This indicates clear stability in the expression of genetic differences among and within sub-races for these compounds.

Examination of the growth data showed that diameter at breast height (DBH) was significantly different between (both data sets) and within (only when the full data set was analysed) sub-races. The significant DBH by site interaction term at the sub-race level was due to poor growth of the St Helens sub-race at the Togari site. We also examined correlations between the chemical traits and DBH and browse damage (Table 3). There were positive phenotypic correlations between DBH and most chemical constituents except for total phenolics which showed a negative phenotypic correlation. No sub-race, nor additive genetic correlations between DBH and chemical traits were detected. Browse damage was phenotypically negatively correlated with macrocarpal, total oil and cineole. These patterns were consistent at the sub-race level for cineole. The opposite pattern was found at the sub-race level for browse and DBH. Browse damage was also positively correlated with total phenolics and nitrogen at the phenotypic level but these patterns were weak.

## Discussion

In this paper we link chemical variation with evolutionary dynamics and ecological interactions across different levels of organisation to demonstrate that (i) significant genetic variation in the expression of many PSMs resides both among and within populations of *E. globulus*, (ii) the expression of this genetic variation is stable across environments, and (iii) that diversifying selection is likely to have acted on some PSMs.

### Hierarchical genetic variation in PSMs and evidence of selection

A major aim of this paper was to estimate genetic parameters (heritabilities and coefficients of additive genetic variance) that inform us of how responsive PSM traits may be to selection [7], [26]. Previous heritability estimates of the ecologically important FPCs in *E. globulus* have been based on broad sense heritability estimates (macrocarpal G 0.51±0.07, sideroxylonal A 0.79±0.04 [51]) but here we report narrow sense heritabilities; which reflect the relative levels of additive variance within populations. Heritability estimates for macrocarpal G were still high (0.48±0.15 and 0.35±0.13 at the Togari and Salmon River sites, respectively) but slightly lower for sideroxylonal A (0.33±0.14 to 0.24±0.12, respectively). Narrow sense estimates for sideroxylonal have also been reported in another eucalypt species where estimates were high (e.g. 0.39±0.12−0.62±0.12[5]). Heritability estimates obviously vary depending on species, populations and environments [17] but what these estimates tell us from these few studies is that these important PSMs are under relatively strong genetic control and there is adequate additive genetic variance within populations for selection. The later is particularly the case for the two FPC compounds, where the coefficient of additive genetic variance (CVa; >15%) is atypically high compared with other traits in *E. globulus* (present study and [52]) other eucalypts [53] and other taxa [7]. This clearly shows these FPC compounds have substantial genetic variation available for exploitation, either through natural or artificial selection, in this species.

Indeed, our examination of differences in the Qst and Fst estimates suggest that sub-race divergence in concentration of foliar PSMs have already evolved through selection. Differences in quantitative trait and neutral molecular estimates have been calculated in a number of plant species to indicate patterns of selection for a range of life history traits [54], [55]. Here, our average Qst estimates for foliar PSMs is significantly higher than the average of Fst estimates for putatively neutral microsatellite loci. For two PSMs, sideroxylonal and cineole, the Qst is significantly higher than the published maximum Fst estimate for putatively neutral microsatellite loci [48], and are amongst the highest Qst values yet estimated for *E. globulus*. While other factors, such as strong genetic correlations amongst traits and correlated responses to selection cannot be dismissed [56], these results argue that natural selection has contributed to sub-race divergence in PSMs in *E. globulus*, and identifies the FPCs (particularly sideroxylonal) and cineole as candidates for traits whose genetic architecture has been shaped by divergent selection [1], [48].

### Possible agents of selection on PSMs in *E. globulus*

Plant secondary metabolites in *E. globulus* may play a role in numerous ecological functions including defence against mammalian herbivores and fire promotion [57], [58]. We have previously suggested that some PSMs in *E. globulus* are under selection by mammalian herbivores because mammalian browsing preferences differ amongst *E. globulus* populations and these differences can be explained, in part, by variation in PSMs among those populations, particularly the FPCs, sideroxylonal and macrocarpal [18], [19]. However, this is the first time that Qst estimates have been calculated for PSMs in this dominant tree species providing an independent line of evidence that these traits have been under divergent selection. Here, we also link these estimates with observed negative phenotypic correlations between field browsing by mammalian herbivores, macrocarpal G and cineole (however, negative correlations between browsing and sideroxylonal were not significant in this study). There were also negative phenotypic and additive genetic correlations between field browsing and a surrogate of fitness, stem diameter growth (DBH). In this present study, trees that received less browsing had higher levels of macrocarpal G and cineole and had larger diameters where browsing reduces growth rates as opposed to browsers selecting smaller trees [59]. In support of this surrogate fitness measure, longer-term genetic studies in similar competitive plantings have shown *E. globulus* survival is size dependent [52]. Given the relationships with browsing, FPCs (e.g. [18], [19]; and this present study for macrocarpal) and growth, the idea that cineole acts as an olfactory cue to the FPCs [60] and the genetic data presented in this present study, there is clear evidence that PSMs in *E. globulus* are under selection and that that mammalian herbivores may play a role. The correlated nature of the compounds [19], however, makes it less clear exactly which traits may be under direct selection.

Obviously, fitness estimates are difficult to obtain in a long lived tree species and hence surrogates of fitness (such as DBH and reproductive output at a snapshot in time) are often our best option. While we have only assessed DBH in this current study, we have shown that repeated browsing by mammalian herbivores greatly reduces individual plant reproductive fitness (number of buds and capsules) at 10 years of age compared to those plants that were not browsed [61]. In this current paper, there is also little evidence of a trade-off between PSM production and growth [62], [63], although we cannot discount costs at different life stages of the plant [64]. Most phenotypic correlations between PSMs and DBH were significant and positive (apart from total phenolics) and we did not detect any significant negative correlations at the sub-race or additive levels.

### Population divergence and geographic patterns of PSMs in *E. globulus*

Broad-scale population divergence in the expression of PSMs in this study appear to follow some clear geographic patterns. The two mainland Australian regions, the south-western Victorian sub-races (Eastern and Western Otway) and the south-eastern Victorian sub-races (Strzelecki and Coastal Plains) show divergent patterns of PSM expression. If one region is high in concentration of PSM then the other region is low (evident for sideroxylonal A, macrocarpal G and total oils). These two distinct groupings are also reflected in the UPGMA clustering of sub-races based on variation in PSMs presented in this current paper and also in Wallis et al [22] where the two Victorian regions were shown to be dominated by different ’chemotypes’. Molecular studies have shown that these two mainland regions also differentiated on nuclear microsatellite markers [48], [49], chloroplast DNA haplotypes frequencies (Freeman et al, 2001) and recent diversity arrays technology markers (DArT) [65]. Hence, genetic differentiation in these PSM traits also appears to be associated with reduced gene flow between these regions.

Another clear pattern is the steep latitudinal cline in most PSMs (sideroxylonal A, total oils, cineole and total phenolics) from Flinders Island, Southern Furneaux through to the north-eastern Tasmanian sub-race of St Helens and where Flinders island groups more closely to the south-eastern Victorian sub-races. Clinal patterns in quantitative traits have become a point of interest in recent literature due to probable effects of large scale environmental variation (e.g. climate change effects) on local adaptation of populations [66]. Our clinal trends in chemistry are consistent with that reported across the island group by Wallis et al [22] which appears to coincide with a barrier to seed dispersal suggested by the north-eastern Tasmanian populations lacking a specific chloroplast haplotype which is common in eastern Victoria and present on Flinders Island and the Southern Furneaux [67]. However, while there may be a barrier to seed dispersal, there is evidence of pollen flow [68] supporting the steep cline evident in this study.

Finally, based on a chloroplast DNA study, Freeman et al. [67] also postulated a recent ‘western-link’ between the mainland and Tasmania, from the Otways, through King Island and down the west coast and moving up the east coast of Tasmania. The genetic-based clinal differentiation in PSMs reported in Wallis et al. [22] supported this pattern and suggests a clinal loss of plant PSM defences along this gradient. This appears to have culminated in the evolution of the least defended north-eastern Tasmania populations [18], [19]. While our study argues for the role of disruptive selection and marsupial herbivory in shaping these sub-race patterns of differentiation (but to date we have no evidence of differential selection pressure), the steep cline across the Furneaux Islands potentially represents a dynamic zone of secondary contact and pollen-mediated gene flow between the highly defended, eastern mainland and least defended, north-eastern Tasmanian gene pools [22].

### Lack of G×E for PSMs in *E. globulus*

Variability in the genetic expression of traits across environments provides an indication of the relative strength of genetics in affecting ecologically important traits [66], [69], [70]. This concept is important particularly for plant species that show extended genetic effects and community consequences [71]. To date, only a few studies have examined the plasticity of genetic based variation of PSMs in eucalypts across environments using common pedigrees [5], [72], [73] and few studies have examined G×E interactions of plant chemical defences using a genetic hierarchy in any plant/herbivore system [5]. Here we provide evidence that both among and within population genetic variation in PSMs in *E. globulus* is stable across sites. We did not detect a genotype by environment interaction (G×E) for any of the chemical traits, despite highly significant genetic and site main effects. Previously in nutrient trials, we detected a G×E on mammalian herbivore responses to *E. globulus* in a captive feeding trial, but did not detect a G×E with any of the PSMs we assessed in the seedlings. Additionally, Andrew et al. [5] demonstrated a G×E interaction on sideroxylonal in *Eucalyptus tricarpa* at the population level, but not within populations. In some recent work, we manipulated CO_2_ levels in the glasshouse with different populations of two eucalypt species, *E. globulus* and *E. pauciflora,* and found that CO_2_ had minimal effect on the expression of PSMs and no G×E was detected [73].

What is becoming apparent in eucalypt systems is that while the levels of PSMs may change with environment, the relative differences amongst genotypes remains stable. Low or absent G×E for eucalypt PSMs has important ecological and applied management implications. If these genetic based traits that influence community phenotypes are stable across environments, the role of plant genotype in structuring communities as observed in *E. globulus* [20], is strengthened and these genotypic differences may be relatively stable under global environmental change such as increasing atmospheric CO_2_. Additionally, as many eucalypt species are important commercial forestry species (*E. globulus* is the most widely planted hardwood species in temperate regions worldwide) the knowledge that genotypes may be stable in the expression of PSMs across different plantation sites give confidence to tree growers for artificial selection for these traits in order to reduce losses from pest damage [74].

## Supporting Information

Table S1 **Table of pair-wise Mahalanobis distances amongst** ***E. globulus*** **sub-races and their significance.** The Mahalanobis distances were calculated using the PROC DISCRIM procedure of SAS and data set comprising the native-forest family means (calculated across sites) for the six chemical components analysed.
(DOC)
