# Supplementary material for: Chemical Variation in a Dominant Tree Species: Population Divergence, Selection and Genetic Stability across Environments
Source: PLoS One. 2013 Mar 20;8(3):e58416. doi: 10.1371/journal.pone.0058416 (PMC3603948; doi:10.1371/journal.pone.0058416)
Supplement: Table S1 — Table of pair-wise Mahalanobis distances amongst E. globulus sub-races and their significance. The Mahalanobis distances were calculated using the PROC DISCRIM procedure of SAS and data set comprising the native-forest family means (calculated across sites) for the six chemical components analysed. (DOC) [file pone.0058416.s001.doc]

Table S1.

|  |  |  |  |  | | North- | |  | | South- |  |  |  |  |
| --- | --- | --- | --- | --- | --- | --- | --- | --- | --- | --- | --- | --- | --- | --- |
|  | Coastal | Eastern | Flinders | King | | eastern | | Recherche | | eastern | Southern | St | Strzelecki | Western |
|  | Plain | Otways | Island | Island | | Tasmania | | Bay | | Tasmania | Furneaux | Helens | Ranges | Otways |
| Eastern Otways | 20.30*** |  |  |  | |  | |  | |  |  |  |  |  |
| Flinders Island | 7.50* | 24.65*** |  |  | |  | |  | |  |  |  |  |  |
| King Island | 12.25*** | 13.91*** | 10.28* |  | |  | |  | |  |  |  |  |  |
| North-eastern Tasmania | 8.63** | 8.69** | 12.46** | | 2.30 |  | |  | |  |  |  |  |  |
| Recherche Bay | 11.42** | 10.44* | 16.59** | | 2.08 | | 0.85 |  | |  |  |  |  |  |
| South-eastern Tasmania | 14.44*** | 13.17*** | 17.89*** | | 2.45 | | 2.89 | | 0.97 |  |  |  |  |  |
| Southern Furneaux | 3.81 | 13.42*** | 8.06* | | 3.00 | | 1.82 | | 2.53 | 3.71 |  |  |  |  |
| St Helens | 26.98*** | 21.86*** | 42.81*** | | 16.55*** | | 10.32*** | | 8.77* | 12.83*** | 16.05*** |  |  |  |
| Strzelecki Ranges | 3.26 | 32.81*** | 10.86* | | 16.86*** | | 12.81*** | | 15.94** | 18.36*** | 7.09* | 29.78*** |  |  |
| Western Otways | 19.89*** | 2.58 | 28.59*** | | 13.20*** | | 6.92* | | 8.25* | 12.86*** | 12.80*** | 11.68*** | 31.23*** |  |
| Western Tasmania | 16.42*** | 16.62*** | 20.98*** | | 4.79 | | 3.72 | | 2.11 | 1.39 | 5.57*** | 11.97*** | 17.50*** | 15.46*** |

*significant at P < 0.05; **significant at P < 0.01; ***significant at P < 0.001
